# Supplementary material for: Quantitative Analysis of the Seminal Plasma Proteome in Secondary Hypogonadism
Source: J Clin Med. 2019 Dec 3;8(12):2128. doi: 10.3390/jcm8122128 (PMC6947469; doi:10.3390/jcm8122128)
Supplement: Supplementary file 1 [file jcm-08-02128-s001.pdf]

	Hypogonadic Patients (N=7)	Range values
Age	41.35 ± 12.25	
Testosterone (T)	1.72 ± 0.76	2.5-8.4 ng/ml
Estradiol (E2)	24.06 ± 3.44	15-44 pg/ml
SHBG	18.68 ± 5.31	16-80 nmol/L
FSH	1.63 ± 0.94	1.0-8.0 mU/ml
LH	0.84 ± 0.35	2.5-10.0 mU/ml
Seminal volume	1.48 ± 0.98 ml	
Sperm concentration	3.54 ± 4.54 x 10^6/ml	
Total sperm motility	9.36 ± 11.32 %	
Normal morphology	3.15 ± 3.07 %	

Supplementary Table 1. Clinical and hormonal parameters of the population of 7 additional hypogonadic patients furtherly added in western blot analysis.

Supplementary Figure 1. Picture of all gels after enhanced chemiluminescence, all pictures of membranes after red ponceau staining and western blot membrane with detected bands.

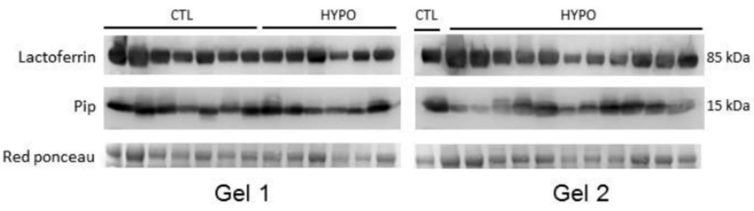

Gel 1

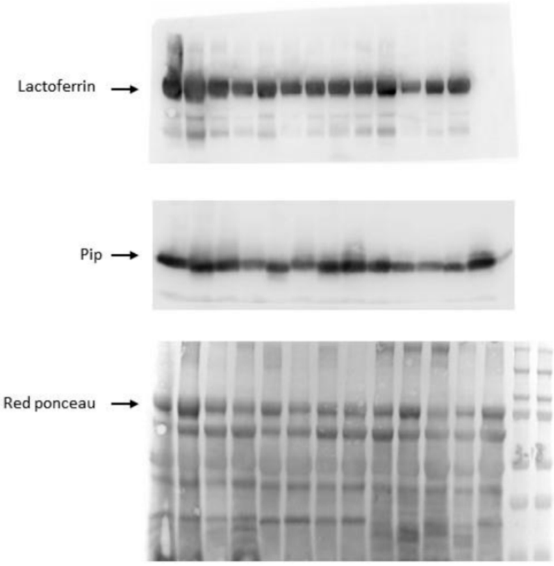

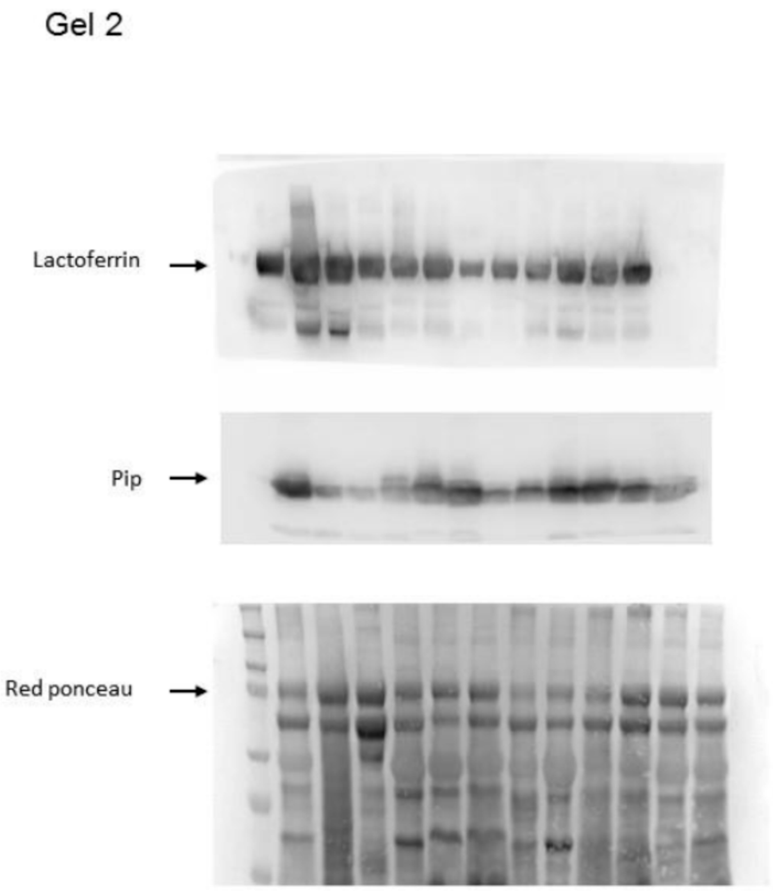

Supplementary Figure 2. Quantification analysis of western blot

Quantification WB Figure 1

red ponceau

gel 1

Number	Vol. %	Volume	Height	Area
ctl	0.793	3.62E+08	42740	15750
ctl	1.313	6.01E+08	49170	18270
ctl	0.98	4.48E+08	38200	17514
ctl	1.015	4.64E+08	34648	18522
ctl	1.049	4.8E+08	39229	17010
ctl	1.019	4.66E+08	34248	18774
ctl	1	4.57E+08	38098	16632
hypo	1.119	5.12E+08	37145	19404
hypo	1.004	4.59E+08	40029	16506
hypo	1.224	5.6E+08	46112	18396
hypo	0.935	4.28E+08	30544	19530
hypo	0.681	3.11E+08	29776	16632
hypo	0.988	4.52E+08	36782	19908

gel 2

Number	Vol. %	Volume	Height	Area
ctl	1	3.61E+08	31304	19728
hypo	1.744	6.29E+08	45645	21646
hypo	1.784	6.44E+08	48232	20824
hypo	1.535	5.54E+08	42058	19865
hypo	1.599	5.77E+08	43738	20276
hypo	1.734	6.25E+08	46377	20961
hypo	1.186	4.28E+08	32788	17673
hypo	1.267	4.57E+08	36225	18358
hypo	1.359	4.9E+08	37169	19317
hypo	1.757	6.34E+08	46632	20276
hypo	1.472	5.31E+08	43717	18632
hypo	1.388	5.01E+08	37631	20139

**ctl**	0.793
	1.313
	0.98
	1.015
	1.049
	1.019
	1
**hypo**	1.119
	1.004
	1.224
	0.935
	0.681
	0.988
	1.744
	1.784
	1.535
	1.599
	1.734
	1.186
	1.267
	1.359
	1.757
	1.472
	1.388

Lactoferrin

gel 1

Number	Vol. %	Volume	Height	Area
ctl	1.248	1.1E+08	31563	6241
ctl	1.195	1.06E+08	22802	6715
ctl	1.082	95741629	24224	6715
ctl	1.003	88678987	26280	6715
ctl	1.158	1.02E+08	30653	5925
ctl	0.976	86358556	25079	6873
ctl	1	88456183	26269	6557
hypo	1.132	1E+08	26410	7031
hypo	1.031	91230925	27439	6478
hypo	1.301	1.15E+08	38866	6320
hypo	0.699	61860109	25049	6399
hypo	0.983	86981185	31127	5925
hypo	1.19	1.05E+08	31026	7189

gel 2

Number	Vol. %	Volume	Height	Area
ctl	1	93230795	35311	6399
hypo	0.971	90534382	22602	6952
hypo	0.951	88685095	22763	6636
hypo	0.82	76419943	23488	6083
hypo	0.779	72642837	21223	6399
hypo	0.934	87031647	23527	6794
hypo	0.533	49735048	20547	6162
hypo	0.689	64252216	23030	6399
hypo	0.602	56092155	24576	6320
hypo	0.926	86333689	25850	6794
hypo	0.729	67996468	20294	6636
hypo	1.018	94890968	29794	6952

		Lactoferrin/red ponceau	M	sd	T
**ctl**	1.248	1.57	1.091124	0.224616	0.009613
	1.195	0.91			**
	1.082	1.10			
	1.003	0.99			
	1.158	1.10			
	0.976	0.96			
	1	1.00			
**hypo**	1.132	1.01	0.725805	0.306924	
	1.031	1.03			
	1.301	1.06			
	0.699	0.75			
	0.983	1.44			
	1.19	1.20			
	0.971	0.56			
	0.951	0.53			
	0.82	0.53			
	0.779	0.49			
	0.934	0.54			
	0.533	0.45			
	0.689	0.54			
	0.602	0.44			
	0.926	0.53			
	0.729	0.50			
	1.018	0.73			

	Lactoferrin	sd
**CTL**	**1**	**0.224616**
**HYPO**	**0.67**	**0.306924**

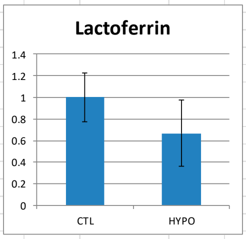

CTL/IPO

1.50

pip

gel 1

Number	Vol. %	Volume	Height	Area
No 1	1.262	2.73E+08	50915	16714
No 2	1.345	2.91E+08	47547	13426
No 3	1.04	2.25E+08	43872	13152
No 4	0.686	1.48E+08	32656	12467
No 5	0.915	1.98E+08	41238	12330
No 6	0.819	1.77E+08	35675	12741
No 7	1	2.16E+08	45424	11645
No 8	1.27	2.75E+08	48268	13289
No 9	0.957	2.07E+08	46356	12467
No 10	0.657	1.42E+08	38891	11782
No 11	0.602	1.3E+08	34104	13152
No 12	0.698	1.51E+08	41207	12467
No 13	1.298	2.81E+08	51267	14111

gel2

Number	Vol. %	Volume	Height	Area
No 1	1	3.04E+08	45702	15892
No 2	0.35	1.07E+08	29497	11782
No 3	0.202	61612890	16378	11508
No 4	0.387	1.18E+08	21001	12878
No 5	0.696	2.12E+08	35287	12878
No 6	0.861	2.62E+08	42588	14111
No 7	0.337	1.03E+08	28632	10686
No 8	0.486	1.48E+08	35763	12330
No 9	0.81	2.46E+08	46174	12604
No 10	0.849	2.58E+08	42997	13426
No 11	0.668	2.03E+08	38490	13426
No 12	0.51	1.55E+08	25508	16440

		pip/red ponceau	M	sd	T
**ctl**	1.262	1.59	1.004125	0.292664	0.007644
	1.345	1.02			**
	1.04	1.06			
	0.686	0.68			
	0.915	0.87			
	0.819	0.80			
	1	1.00			
**ipo**	1.27	1.13	0.569031	0.342924	
	0.957	0.95			
	0.657	0.54			
	0.602	0.64			
	0.698	1.02			
	1.298	1.31			
	0.35	0.20			
	0.202	0.11			
	0.387	0.25			
	0.696	0.44			
	0.861	0.50			
	0.337	0.28			
	0.486	0.38			
	0.81	0.60			
	0.849	0.48			
	0.668	0.45			
	0.51	0.37			

	pip	sd
**CTL**	**1**	**0.292664**
**HYPO**	**0.57**	**0.342924**

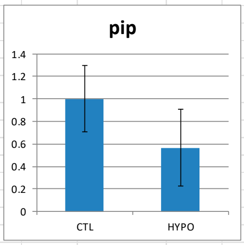

CTL/IPO

1.76
